# Reproductive concessions between related and unrelated members promote eusociality in bees

**DOI:** 10.1038/srep26635

**Published:** 2016-05-23

**Authors:** Aline C. R. Andrade, Elder A. Miranda, Marco A. Del Lama, Fábio S. Nascimento

**Affiliations:** 1 Laboratório de Comportamento e Ecologia de Insetos Sociais - Departamento de Biologia - FFCLRP – Universidade de São Paulo - Av. Bandeirantes, 3900, Ribeirão Preto - SP, Brasil; 2 Laboratório de Genética Evolutiva de Himenópteros, Departamento de Genética e Evolução, Universidade Federal de São Carlos, São Carlos, SP, Brasil

## Abstract

Animal societies exhibit remarkable variation in their breeding strategies. Individuals can maximize their fitness by either reproducing or by helping relatives. Social hymenopterans have been key taxa for the study of Hamilton’s inclusive fitness theory because the haplodiploid sex-determination system results in asymmetric relatedness among breeders producing conflict over the partitioning of reproduction. In small cooperative groups of insects, totipotent individuals may maximize their inclusive fitness by controlling reproduction despotically rather than helping their relatives. Here, we demonstrate that the dominant females of the primitively eusocial bee *Euglossa melanotricha* (Apidae: Euglossini) control reproduction, but concede part of the reproductive output with their related and unrelated subordinates. As expected, a dominant female capitalizes on the direct reproduction of related subordinates, according to her interests. We found that reproductive skew was positively correlated with relatedness. The concessions were highly reduced in mother-daughter and sibling nests (relatedness *r* ± s.d. = 0.54 ± 0.02 and 0.79 ± 0.02, respectively) but much more egalitarian in unrelated associations (*r* = −0.10 ± 0.01). We concluded that reproductive skew in these primitively eusocial bees is strongly related to the genetic structure of associations, and also that females are able to assess pairwise relatedness, either directly or indirectly, and use this information to mediate social contracts.

The cooperation of organisms to form a higher level of biological organization represents a major evolutionary transition^1^. Maintenance of a stable social group demands specific benefits to offset the costs incurred by individuals that help others reproduce. Individuals may maximize their inclusive fitness by controlling reproduction despotically or helping relatives. Kin selection predicts that animals will act in ways that tend to maximize their inclusive fitness^2^.

In social hymenopterans, relatedness asymmetries between nestmates produce conflicts of interest as individuals simultaneously attempt to maximize their own reproduction^2^. In small insect societies, the most obvious potential conflict between breeders concern the partitioning of reproduction (reproductive skew) in groups lacking morphologically differentiated castes, where more than one individual is capable of reproduction^3^. How conflicts are resolved depends on the payoffs of the different reproductive strategies to each individual^4^,^5^.

Reproductive skew theory has provided an important framework for understanding these strategies^6^,^7^,^8^,^9^,^10^. This theory is particularly interesting because it is relatively simple, comprising some aspects of the payoffs involved in alternative social contexts and the mediation of these payoffs such as competitive ability and relatedness^8^. The models based on skew theory attempt to discuss the skew based on the trade off of reproductive benefits, the result of which is shaped by a number of different social and ecological factors, including relatedness, resource-holding potential, group productivity and constraints on independent breeding^11^. The theory provides a convincing explanation of how and why conflicts are resolved, and has been suggested as a general theory of social evolution^8^.

Previous studies have shown that a positive or negative relationship between skew and relatedness could be used to support transactional or tug-of-war models^12^,^13^,^14^. However, the generality of each model is restricted by their assumptions. Transactional models assume that a single dominant individual has control over group membership and the fraction of total group reproduction obtained by the subordinate breeder^15^. The dominant breeder maximizes her own fraction of reproduction at the expense of a related subordinate, but concedes just enough reproductive output to the subordinate to make it favorable for this individual to stay in the group. As an unrelated subordinate lacks this indirect benefit of staying, the dominant female must grant her a share of direct reproduction to maintain the association (individuals can negotiate based on the threat of group dissolution - “outside option”)^8^. Thus, one of the main predictions of the model is that reproductive skew will be high when relatedness between breeders is high^15^,^16^,^17^. In the tug-of-war models, neither individual has control over the allocation of reproduction^8^,^12^,^18^ (individuals can negotiate based on the threat of costly competition – the inside option)^8^. In contrast with concessions models, tug-of-war models predict the absence of a relationship between relatedness and skew^18^. This assumption of costly competition by both individuals impedes the evolution of more efficient form of reproductive sharing.

The solution to this problem can be the association of the assumptions of the models of reproductive skew using Hamilton’s rule to predict the conditions under which the assumptions of major classes of models (transactional and tug-of-war) consider^8^. Therefore, synthesizing the transactional and tug-of-war models, it is possible to determine the conditions under which individuals will negotiate based on their options to leave or to stay^8^.

A previous study showed that females of the allodapine bee *Exoneura robusta* are able to assess pairwise relatedness, either directly or indirectly, and use this information to mediate ovarian development^19^. This study suggests a path for future developments in skew theory, drawing attention to what has been widely considered to be an obscure point: the ability of individuals to acquire and process the types of information required for models of skew theory to function^19^.

*Euglossa melanotricha* nests are usually multivoltine. Solitary females found new nests or can re-use inactive nests by mixing new with old resin to build the new cells. The process of nest re-use can be initiated when two newly-emerged females remain in their natal nest and one begins to reproduce (Fig. 1). Previous studies have shown that the multifemale societies of this orchid bee are usually formed by a mother and her daughters (matrifilial nests), sisters (full sibling nests) or usurpers and resident females (unrelated female nests)^20^,^21^,^22^. Different from other bee species, all *E. melanotricha* females can mate, but egg laying is regulated by the dominant’s behaviour and chemical signalling^22^. These behavioural features provide a rare opportunity to test predictions of the skew reproductive theory.

Here, we predicted that dominant *E. melanotricha* females may do better to concede a small and cheap share of reproduction rather than enter into an escalated contest with a highly motivated subordinate. We evaluated the benefits of direct and indirect reproduction related to the genetic structure within the nests. In this primitively eusocial bee, dominant females control reproduction and capitalize on the direct reproduction of related and unrelated subordinates according to their interests.

## Results

### Microsatellite data analysis

No significant linkage disequilibrium between loci was detected. For the analysis of allelic variation at each locus, we genotyped males (n = 159) and pooled these data with those of unrelated females (n = 54). Genetic diversity estimates are given in Table S1 – Supplementary information. The expected heterozygosity (He) of markers ranged from 0.806–0.926 and we found between nine and 17 alleles per locus. Also, the marker was clearly inherited in a strictly Mendelian manner within families of bees. As a result of the high variability of our markers, the population-wide probability of genetic non-detection of a second male fathering offspring among progeny genotypes was very small; the non-detection error (dp) varied from 0.002–0.00005. No evidence was detected of null alleles, scoring errors due to stuttering, or major allele dropout.

### Conflict resolution by reproductive concessions between totipotent females

**Figure 1:**
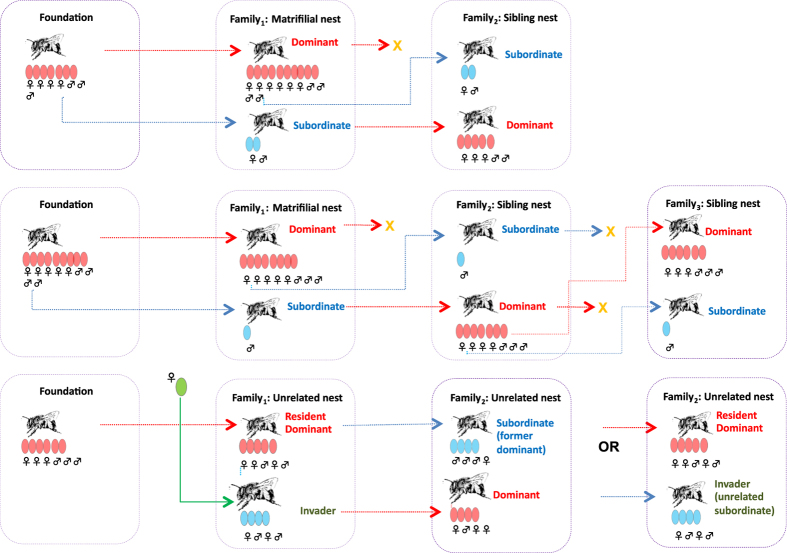
Life cycle and types of nest associations of *Euglossa melanotricha*. The cycle begins with a solitary nest founded by a single female. Two possible options of the first cohort females (blue label) are shown. Newly emerged females stay in the nest and become subordinates. They can inherit the nest when dominant dies or disappears or reactivate a nest with sisters. Subordinates will perform typical worker activities such as foraging and nest maintenance. However, subordinates share partially reproduction with dominants. Unrelated invaders can overthrow dominance when they are larger than residents or become subordinate helpers. Red arrows represent the routes of dominants and blue arrows represent the routes of subordinates. Letter X represents females dying or disappearing from the nest.

In the present study, the mean ± SD duration of the re-use process (from the time a female started foraging for resin to her final oviposition) was 46.4 ± 14.9 days (range 18–79 days, n = 30 nests). Following re-use, the females remained in the nest without engaging in any further outside activities. This period of inactivity lasted from 15–63 days (34.1 ± 11.7 days). The mean ± s.d. interval between emergence of one adult and another was 2.47 ± 0.67 (range 2–5 days, n = 30 nests). The reproductive dominance among females is determined by aggressive interactions and by egg removal (supplementary videos), which results in an age-based social hierarchy^11^. When the dominant bee dies or disappears, she will be replaced by one of the older subordinates (Fig. 1).

At the population level, 100% of the first and second emergences, and approximately 80% of the third emergences of newly emerged females were produced by the dominant bees (relatedness = 0.5 ± 0.04; n = 18 families), but relatedness with the dominant female decreased significantly (*D* = 0.6; *p* < 0.01; n = 18 families; see Table S2 – Supplementary information) in the subsequent (fourth to seventh) emergences. The genetic relatedness between subordinates and female offspring remained close to 0.5 over all seven episodes of emergence, however. At the colony level, this is an incentive for the older, higher-ranked subordinates to remain in the nest, while the younger, lower-ranked subordinates will have no opportunities to reproduce, and will frequently leave the nest.

**Figure 2:**
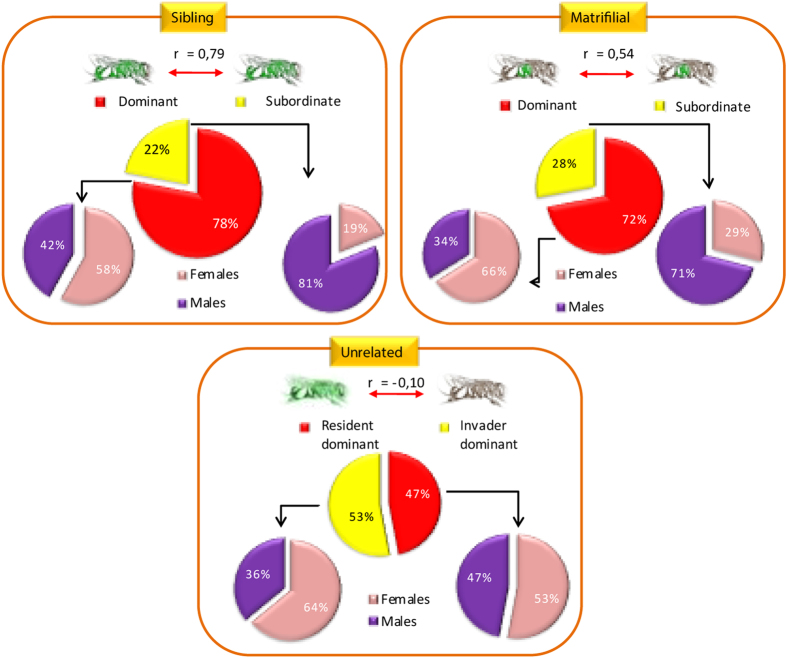
Sociogenetic structure showing the output of social contracts. The genetic relatedness between the females in each family group and the sex ratio of the offspring of these females was determined by genotyping. Dominant and subordinate females were recognized through behavioural interactions, egg laying and oophagy. Consistent with the predictions of reproductive concession regulated by dominants, dominant bees in the sibling nests reared 34 females and 25 males (**a**) χ^2^_1_ = 1.373, p > 0.05), while subordinate sisters raised mainly males (9 females to 11 males; χ^2^_1_ = 4.45, p < 0.05). In matrifilial nests, dominants invested in a female-biased reproductive sex ratio (**b**) 36 females to 19 males, χ^2^_1_ = 5.25, p < 0.05), whereas their daughters produced a male-biased ratio (6 females to 15 males; χ^2^_1_ = 5.76, p < 0.05). The overall reproductive output of matrifilial nests was 42 females to 34 males, which does not deviate significantly from a 1:1 ratio (χ^2^_1_ = 0.02 p > 0.05). In unrelated associations, on the other hand, reproductive output was more evenly balanced (62 females to 50 males), with invaders producing 32 females and 23 males (**c**) χ^2^_1_ = 1.47, p > 0.05) and dominant resident bees, 30 females and 27 males (χ^2^_1_ = 0.15, p > 0.05).

Reproductive output was affected significantly by the class of females (GLM: Wald’s test = 13.54; d.f. = 1, *p* = 0.004) and its interaction with the type of nest (matrifilial, sibling or unrelated nests) (GLM: Wald’s test = 7.05; d.f. = 2, *p* = 0.029) (Table S3 – Supplementary information). Specifically, dominants produced more female offspring in both matrifilial and sibling nests than in unrelated nests (Mean ± s.e.: matrifilial = 6.55 ± 0.42; sibling = 6.11 ± 0.42; unrelated = 4.58 ± 0.36; Fig. 2), while subordinates produced more offspring when associated with an unrelated dominant (Mean ± s.e.: matrifilial = 2.33 ± 0.42; sibling = 1.22 ± 0.42; unrelated = 4.75 ± 0.37).

**Figure 3:**
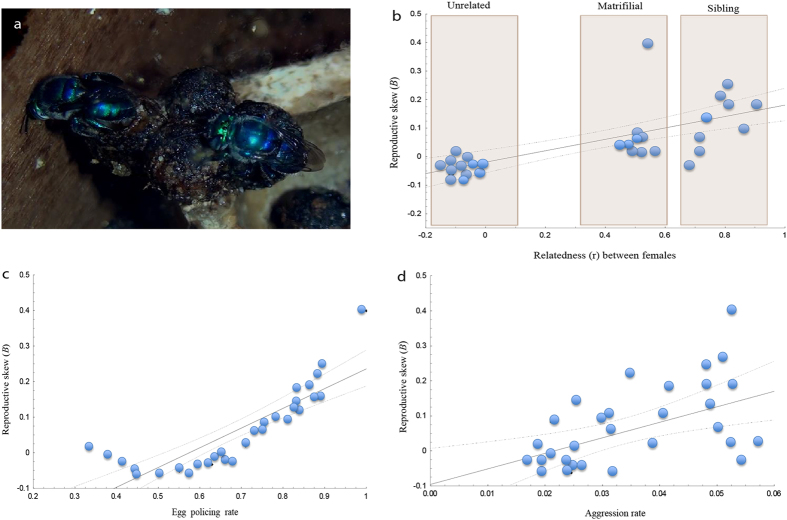
Skew in *Euglossa melanotricha*. (**a**) An interaction between adult females (dominant and subordinate). The dominant female (right) is monitoring the subordinate female (left) during oviposition. The graphs show the coefficients of regression between the skew (*B*) and the following variables: (**b**) relatedness (*r*), (**c**) oophagy rates, and (**d**) aggression rates between reproductive females. The data include unrelated, matrifilial and sibling nests, and demonstrate a significant positive correlation between skew and all three variables (Pearson’s r = 0.88, p < 0.0001; r = 0.79, p < 0.0001; r = 0.65, p < 0.0001 for (**b**–**d)**, respectively; n = 30 families). The solid line indicates the linear regression with its 95% confidence interval indicated by the dotted outliers.

Across all families, reproductive skew was positively correlated with the degree of relatedness (Pearson’s product correlation *r* = 0.88, n = 30, *p* < 0.0001; Fig. 3), as well as the frequency of egg removal and aggressive acts (Pearson’s *r* = 0.79, *p* < 0.0001 and *r* = 0.65, p < 0.0001, respectively; Fig. 3). However, a multivariate analysis between reproductive skew and behavioural variables revealed that relatedness was the most significant variable to explain the reproductive conflict within nests (Table S4 – Supplementary information). Coercion mechanisms were typically mediated by the relative reproductive roles of the females in the nest rather than body size or ovarian development^10^. Our results confirmed that dominance in *E. melanotricha* is expressed through direct aggression and active oophagy, with a clear division of labour and hierarchy among nestmates, although these behavioural traits do not result in the suppression of ovarian function in subordinates (Table S5 – Supplementary information).

Reproductive dominance was more intense in highly skewed families (sibling and mother-daughter associations). This led frequently to the suppression of reproduction in the subordinates by a more despotic dominant female^15^. Indeed, the removal of eggs was also determined by the degree of relatedness between breeders (*F* = 58.06, d.f. = 27, *p* < 0.0001; Table S5 – Supplementary information). Dominant females ate 84% of the eggs (59 of 70 eggs) laid by their sisters in sibling nests, 72% of the eggs (55/76) laid by their daughters in matrifilial nests, and 51% of the eggs (57/112) laid by subordinates in unrelated associations. Overt aggressive behaviours occurred most frequently in sibling and mother-daughter nests than in those with unrelated females (*F* = 4.01, d.f. = 27, *p* < 0.028; Fig. 3 and see also Table S5 – Supplementary information). As predicted, dominance-related aggression appeared to be more prevalent in social groups that are under strong ecological constraints but, counter-intuitively, comprised of close relatives^17^,^18^.

### Direct and indirect fitness

**Figure 4:**
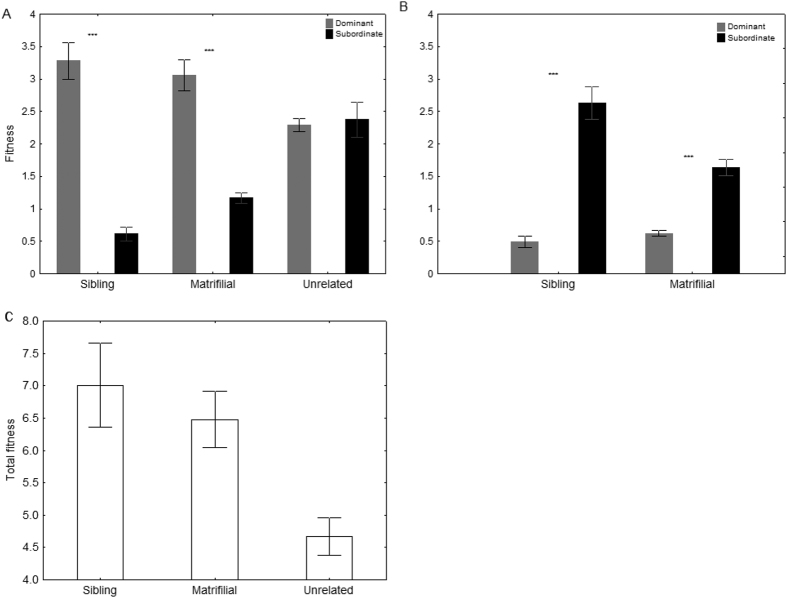
Fitness of multifemales nests. **(A**) Average direct fitness of dominants and subordinates in different types of associations. (**B**) Indirect fitness of females in sibling and matrifilial nests. (**C**) Inclusive fitness (direct + indirect) of all females in related and unrelated associations. Asterisks indicate pairwise comparison *p* < 0.001.

Dominant females had much higher direct fitness in both types of related associations than in unrelated nests (Fig. 4A), as they shared less direct reproduction with their subordinates (see above). However, mean estimated indirect fitness showed that subordinates in highly related associations (full sisters and daughters) had higher benefits than their dominants (Fig. 4B). Total fitness (inclusive) of related females was significantly higher than that between unrelated females (Fig. 4C). To verify the benefits of cooperative versus solitary nesting, we removed the subordinate females from 30 nests. Immediately following the absence of the subordinates, the dominant females continued to provision new cells, but at a significantly lower mean rate (4.8 ± 1.19 new cells) than in the presence of subordinates (8.4 ± 1.97 new cells: Wilcoxon two-sample test: *T* = 3, *n* = 14 nests in 30 families, *p* = 0.002). Without the subordinates, the dominant females re-initiated foraging trips, with the mean number of trips reaching 2.03 ± 0.45 trips/h (*n* = 14 nests). The significant reduction in reproductive output following the removal of subordinates indicates that the division of labour has clear benefits for both breeders and non-breeders.

## Discussion

Our results demonstrate that the reproductive output of subordinates in *E. melanotricha* was affected by their degree of relatedness with the dominant female. Unrelated subordinates produced 53% of all offspring, whereas subordinate daughters contributed 28%, and sisters, 22%. Sociality in *E. melanotricha* may be mediated by the interplay between the relatedness of breeders and the relatively high probability that a subordinate will eventually inherit the dominant, egg-laying role. The magnitude of the reproductive skew depended on the relatedness between group members. Taken together, these results support the predictions of the transactional model, in which the dominant female controls reproductive output and allows the subordinates to reproduce only as far as necessary to prevent them from leaving the nest to reproduce independently^8^,^10^,^17^. In this case, the dominant female would be expected to capitalize on the direct reproduction of the subordinates according to the asymmetry of their relatedness. On the other hand, related subordinates obtain greater indirect fitness by raising the dominant’s offspring^2^,^9^. However, we found that the relative reproductive output and the female-biased sex ratio of the offspring both decreased in associations in which the females were more closely related.

In fact, dynamic skew models also consider how delayed benefits accruing from remaining in the group may affect reproductive skew^8^,^9^,^17^. If survival rates are high, the chances of inheriting dominant status in the future, combined with the reduced success of independent nesting, may explain why subordinates remain as helpers without little or no immediate reproductive incentive^23^,^24^. However, the older *E. melanotricha* subordinates will have the greatest chance of inheriting the principal egg-laying position, and may thus be more willing to help, laying a smaller proportion of eggs, while they wait to inherit the dominant position. In *Polistes* paper wasps, nest inheritance can explain the presence of unrelated helpers - subordinate helpers produced more direct offspring than lone breeders, some while still subordinate, but most after inheriting the dominant position. Thus, while indirect fitness obtained through helping relatives has been the dominant paradigm for understanding eusociality in insects, direct fitness is vital to explain cooperation^25^,^26^,^27^,^28^,^29^.

The skew theory models also predict that dominance-related interactions should be more common in high-skew societies, in which the greater disparity in relative breeding success should motivate subordinates to challenge the dominant female, with the dominant female thus being more likely to invest more effort in suppressing subordinates^30^,^31^. When skew is low, the potential reproductive rewards for challenging and replacing the dominant female will be much smaller, so interactions between breeders will be expected to be more moderate. In other words, dominance-related aggression is expected to be more prevalent in social groups that are under strong ecological constraints and, counter-intuitively, comprise close relatives^32^,^33^. In *E. melanotricha*, reduced relatedness was also reflected in fewer disputes, favouring weaker (or no) dominance behaviour, while high levels of relatedness were reflected in conspicuous reproductive conflict and intense dominance-related coercion among group members. We confirmed that increased relatedness between breeders results in a higher skew, which in turn makes conflict more likely^30^. Thus, according to the predictions of a ‘social contract’ inherent to the transactional models^8^,^9^, a single dominant female will assume the control of group membership but will share just enough reproduction to make it favourable for subordinates to remain in the nest. As an unrelated subordinate will lack any indirect benefit, the dominant female must concede a greater share of direct reproductive output in order to guarantee the association. An alternative hypothesis is that the skew of reproductive dominance is determined by selfish competition between group members, as predicted by tug-of-war models^8^,^15^,^18^.

Several studies have shown a relationship between skew and relatedness^8^,^11^,^12^,^13^,^14^. In the facultative social wasp *Microstigmus nigrophthalmus*^14^, reproductive skew was positively associated with the relatedness of breeders, as well as for cobreeding queens in the ant *Formica fusca*^40^. In contrast, in *Exoneura robusta* and *E. nigrescens*, the available studies^12^,^13^,^19^ have demonstrated a negative relationship between intracolony relatedness and reproductive skew. Indeed, these studies have shown that the ovarian differentiation between queens and secondary breeders, prior to egg-laying, declines with increasing relatedness. Our results support the conclusion that skew is strongly related to relatedness, but not with activation of the ovaries, and also the results indicate that females are able to assess pairwise relatedness, either directly or indirectly, and use this information to mediate social contracts.

Based on the social contract, then, dominant females in matrifilial nests will be predicted to produce a female-biased sex ratio, while the reproductive output of daughters will be male-biased. In this case, subordinates may enhance their fitness by biasing the sex ratio in response to their relatedness with the progeny. An alternative hypothesis would be that the dominant female is unable to control the sexual allocation of reproduction because egg eating will be mutually disadvantageous for both breeders. In this case, group membership and the partitioning of reproductive output will result from the selfish and costly efforts of individuals, in their attempt to guarantee the greatest possible share of group output.

To our knowledge, this is the first study to demonstrate that a social contract between related and unrelated females will modulate reproductive output and promote cooperation in social bees. In particular, the dominant *E. melanotricha* female appears to be able to assess relatedness between nest-mates and selectively remove more or less of the subordinates’ eggs according to its interests. This study provides important new insights for the understanding of social evolution in bees, given the additional evidence for complex forms of social behaviour in a species considered to be primitively eusocial.

## Methods

### Life history

Experiments were carried out from March 2013 and December 2014. We obtained data from focal individuals to compute the proportion of time spent by each bee in a number of common behaviours, and data from all-events sessions to calculate hourly rates of the less common behaviours. We focused on the following four behaviours: (1) Dominance: 30 pairs of females with a stable reproductive relationship (i.e. one individual had been dominant for several weeks) were videotaped continuously using security UV cameras for 24 h (240 days; 5760 h; 12:12 h light: dark cycle). We focused on the performance of the dominant females, including behaviours such as attacking, heading, overflying, pursuing and the cannibalism of the subordinates’ eggs. We computed the rates of dominant acts for each bee, after correcting for the proportion of time bees spent in their nests on a given day. The results were analysed using Wilcoxon’s exact test. (2) Non-dominance or nonaggressive behaviour: active components of non-dominant interactions of the pair (e.g. antennating and approaching). We computed rates of non-dominant interactions for each bee relative to their dominance behaviours. (3) Subordinate behaviour. (4) Other activities: proportion of time that a bee engaged in activities such as foraging trips or remained inside the nest engaging in activities such as resting and cell provisioning for egg laying. We tested the predictions of the reproductive skew models in *Euglossa melanotricha* by evaluating four potential explanatory variables: relatedness, aggressive acts, egg removal, and total reproductive output of cooperative nesting (Fig. 3).

### Behavioural experiments

The study site focused on thirty *Euglossa melanotricha* families (n = 14 nests). We marked the thorax of all the bees in each family using unique spots of quick-drying nontoxic coloured paint (Magic®). These were used for individual identification and monitoring these insects between June 2011 and May 2014. We manipulated the number of females, so that only two females were monitored in each family (the dominant female, n = 30; and their subordinate partners, n = 30). For this, we removed all the additional females (one to three females per trial) that emerged from the nest after we identified the resident pair of individuals. To control for the number of females in each reused nest, we removed the females during this period of inactivity, and waited for the subsequent reoccupation of the nest after the emergence of new females. The offspring of each pair of females were collected as they emerged. The sample size for each family varied between 6 and 15 individuals, including the newly-emerged males and females, and the immature and adult females. Overall, 425 individuals were collected, 159 males and 266 females (Table S6 – Supplementary information). The right middle leg of each individual was stored in absolute alcohol and kept refrigerated at 4 °C for posterior genetic analyses.

### Ovary activation and insemination

All females were dissected under a microscope. Their body size, number of basal oocytes, and insemination status were determined (Table S5 – Supplementary information). The genetic, morphological and behavioural data allowed us to unambiguously distinguish between the dominant and subordinate females, and to determine the maternity of all offspring.

### Genetic analyses and relatedness

The DNA was extracted and amplified using the methods described in Souza *et al*.^34^. Genotyping was carried out after running the amplified DNA fragments in a GE MegaBace-1000 sequencer. Allele sizes were scored using the software MegaBace Fragment Profiler. All adults, brood and immature individuals were genotyped using eleven highly polymorphic microsatellite loci (Table S7 – Supplementary information): seven were originally described in *Euglossa cordata* (Egc 17, Egc 18, Egc 24, Egc 26, Egc 35, Egc 37, Egc 51; ref. 34) and four were designed for *Euglossa annectans* (Ann 03, Ann 04, Ann 24, Ann 37; ref. 35). We tested for linkage disequilibrium between loci within each species with the program GENEPOP^36^ using only the haploid males. Allelic diversity was analysed per site using a standard package of descriptive statistics available in Microsatellite Analyzer^37^. The possibility of null alleles, large allele drop-out and scoring errors was evaluated using micro-checker 2.2.3^38^. Assignment tests of reproductive females as mothers of their offspring were conducted by the visual inspection of the Mendelian segregation of genotypes; all daughters attributed to a mother had to carry a single maternal allele at each locus, and all sons had to carry one of the two maternal alleles at each locus. The Kinship 1.3.1 program^39^ was used to support the determination of the pedigree based on the visual inspection of the genotypes. Comparisons were made with 1,000 pairwise simulations. The kinship (r) of all females was calculated using the kinship function in Kinship 1.3.1 to generate the average value of relatedness between a mother and their offspring of females. We estimated genetic relatedness between reproductive females using the program Relatedness 5.0.8^41^.

### Sex ratio

Offspring sex ratios were estimated by dividing the number of females by the total number of individuals (male + female; refs 42 and 43), and the standard error was calculated for each ratio. To verify potential conflicts, the sex ratio was determined by the proportion of males that emerged from the eggs of the subordinate and dominant females.

### Measuring skew and its correlates

We tested the predictions of the skew models using the *B* index as a measure of skew^44^,^45^, run in the program Skew Calculator 2003^45^. We quantified skew for the overall production of offspring because of the low numbers of male offspring produced. Four potential explanatory variables were quantified to examine their influence on skew: relatedness, aggressive acts, removal of eggs and the productivity benefits of cooperative nesting.

### Fitness estimation

We calculated direct fitness by determining the relatedness of females to their own offspring (r = 0.5, irrelevant of the sex) multiplied by the number of offspring, while the indirect fitness component is the relatedness to the other individuals offspring (r = 0.5* the relatedness estimate, or for the sake of simplicity, 0.75 for siblings, 0.5 for mother daughter and 0 for unrelated) multiplied by the number of offspring of that individual.

### Statistical Analyses

The association between reproductive skew and potential correlates was evaluated using a Pearson correlation coefficient. We used a generalized linear model (GLM) with a binomial error structure and logit link function to verify whether reproductive output was affected by female class (dominant and subordinates), genetic relatedness or the interaction between categorical variables. Nests were entered as random variables^45^. We tested for deviations from a 50% sex ratio per cross and per pair using Chi-square with a Yates correction^46^. The raw data were tested for parametric assumptions with an Anderson-Darling test and Levene’s test. The data were analyzed with parametric tests whenever the assumptions of normality and constancy of variance were met. The data that did not satisfy these assumptions were analyzed with nonparametric tests. All analyses were run in Statistica 10.0 (Statsoft, Tulsa, OK, U.S.A.), with a significance level of α = 0.05.

## Additional Information

**How to cite this article**: Andrade, A. C. R. *et al*. Reproductive concessions between related and unrelated members promote eusociality in bees. *Sci. Rep.* **6**, 26635; doi: 10.1038/srep26635 (2016).

## Supplementary Material

Supplementary Information

Supplementary Video 1

Supplementary Video 2
