# Supplementary material for: Reproductive concessions between related and unrelated members promote eusociality in bees
Source: Sci Rep. 2016 May 23;6:26635. doi: 10.1038/srep26635 (PMC4876382; doi:10.1038/srep26635)
Supplement: Supplementary Information [file srep26635-s1.doc]

Reproductive concessions between related and unrelated members promote eusociality in bees

Aline C. R. Andrade­1*, Elder A. Miranda2, Marco A. Del Lama2 & Fábio S. Nascimento1*

**1** Laboratório de Comportamento e Ecologia de Insetos Sociais - Departamento de Biologia - FFCLRP – Universidade de São Paulo - Av. Bandeirantes, 3900, Ribeirão Preto - SP, Brasil.

2 Laboratório de Genética Evolutiva de Himenópteros, Departamento de Genética e Evolução, Universidade Federal de São Carlos, São Carlos, SP.

*Correspondence and requests for materials should be addressed to A.C.R.A. (e-mail: a.crandrade@usp.br) and F.S.N. (fsnascim@usp.br)

**Supplementary Information**

**Tabela 1** Allelic diversity for *Euglossa melanotricha* families, with the number of genotyped male and female individuals per locus.

| **Loci** | ***n* genotyped individuals** | | **Expected**  **Heterozygosity (He)** | **Mean allele**  **size (bp)** | ***n* observed**  **alleles** | **Allele frequency**  **range** |  |
| --- | --- | --- | --- | --- | --- | --- | --- |
|  |  |  |  |  |  |  |  |
|  | **Males** | **Females** |  |  |  |  |  |
| Egc 17 | 158 | 54 | 0.848 | 223 | 9 | 0.258 – 0.003 |  |
| Egc 18 | 156 | 54 | 0.885 | 183 | 11 | 0.212 – 0.011 |  |
| Egc 24 | 156 | 54 | 0.890 | 203 | 10 | 0.134 – 0.003 |  |
| Egc 26 | 157 | 54 | 0.926 | 172 | 17 | 0.140 – 0.022 |  |
| Egc 35 | 154 | 54 | 0.868 | 164 | 9 | 0.212 – 0.003 |  |
| Egc 37 | 153 | 54 | 0.898 | 196 | 14 | 0.167 – 0.003 |  |
| Egc 51 | 151 | 54 | 0.806 | 226 | 7 | 0.282 – 0.056 |  |
| Ann03 | 155 | 54 | 0.872 | 175 | 12 | 0.180 – 0.008 |  |
| Ann04 | 157 | 54 | 0.859 | 121 | 11 | 0.210 – 0.016 |  |
| Ann24b | 156 | 54 | 0.896 | 174 | 11 | 0.148 – 0.027 |  |
| Ann37b | 155 | 54 | 0.900 | 171 | 12 | 0.129 – 0.005 |  |

**Table 2** Relatedness between the dominant female and the cohorts of offspring that emerged successively in different matrifilial and sibling nests (N = 18).

| **Family group** | **Relatedness (*r*) between Dominant female and offspring** | | | | | | | |
| --- | --- | --- | --- | --- | --- | --- | --- | --- |
|  | **Nest Family** | **Successive emergence:** | | | | | | |
|  |  | **1** | **2** | **3** | **4** | **5** | **6** | **7** |
| **Matrifilial nest** | N5FR1 | 0.51 | 0.55 | 0.56 | 0.22 | 0.50 | 0.51 | 0.53 |
|  | N6FR2 | 0.54 | 0.57 | 0.21 | 0.23 | 0.21 | 0.21 | 0.23 |
|  | N6FR4 | 0.52 | 0.54 | 0.55 | 0.56 | 0.51 | 0.22 | 0.2 |
|  | N7FR1 | 0.59 | 0.59 | 0.51 | 0.55 | 0.23 | 0.22 | 0.19 |
|  | N7FR2 | 0.55 | 0.55 | 0.55 | 0.21 | 0.22 | 0.24 | 0.23 |
|  | N7FR3 | 0.53 | 0.53 | 0.56 | 0.51 | 0.52 | 0.21 | 0.20 |
|  | N8FR1 | 0.56 | 0.67 | 0.51 | 0.52 | 0.24 | 0.20 | 0.21 |
|  | N8FR2 | 0.54 | 0.51 | 0.24 | 0.24 | 0.21 | 0.23 | 0.20 |
|  | N9FR1 | 0.53 | 0.50 | 0.23 | 0.25 | 0.20 | 0.23 | 0.21 |
| **Sibling nest** | N1FR1 | 0.68 | 0.61 | 0.56 | 0.51 | 0.31 | 0.35 | 0.22 |
|  | N1FR2 | 0.52 | 0.55 | 0.55 | 0.35 | 0.34 | 0.38 | 0.33 |
|  | N2FR1 | 0.50 | 0.56 | 0.61 | 0.54 | 0.37 | 0.36 | 0.35 |
|  | N2FR2 | 0.50 | 0.51 | 0.51 | 0.37 | 0.38 | 0.37 | 0.33 |
|  | N2FR3 | 0.53 | 0.64 | 0.56 | 0.52 | 0.33 | 0.33 | 0.36 |
|  | N3FR1 | 0.50 | 0.54 | 0.57 | 0.53 | 0.35 | 0.37 | 0.38 |
|  | N4FR1 | 0.51 | 0.56 | 0.55 | 0.55 | 0.39 | 0.34 | 0.36 |
|  | N6FR1 | 0.56 | 0.52 | 0.51 | 0.34 | 0.33 | 0.36 | 0.35 |
|  | N6FR3 | 0.52 | 0.50 | 0.55 | 0.31 | 0.34 | 0.38 | 0.38 |

**Table 3. Generalized linear model results of offspring production (females and males) of dominant and subordinates in different types of association (matrifilial, sibling and unrelated). Nests entered as a random variable.**

| Effect |  | | | |  |
| --- | --- | --- | --- | --- | --- |
|  | DF | Wald | Log-likehood | Chi-square | *p* |
| Intercept | 1 | 1,58850 | -176,319 |  |  |
| Female class | 1 | 12,43992 | -172,360 | 7,91840 | 0,004893 |
| Genetic structure | 2 | 0,99312 | -171,987 | 0,74563 | 0,688794 |
| Nest | 27 | 10,95916 | -165,704 | 12,56513 | 0,991753 |
| Class*Genetic structure | 2 | 6,64613 | -162,120 | 7,16829 | 0,027760 |

**Table 4. Multiple regression analysis of reproductive skew and behavioural variables**

| Variable |  | | | | | | |
| --- | --- | --- | --- | --- | --- | --- | --- |
|  | β* in | Partial | Semipartial | Tolerance | R-square | t(26) | p-value |
| Aggression | -0,056005 | -0,064071 | -0,040303 | 0,517854 | 0,482146 | -0,327374 | 0,746006 |
| Policing | 0,104581 | 0,141866 | 0,089964 | 0,740002 | 0,259998 | 0,730770 | 0,471454 |
| Relatedness | 0,760078 | 0,616221 | 0,491159 | 0,417569 | 0,582431 | 3,989625 | 0,000480 |

**Table 5** Number of aggressive acts and oophagy in the families groups of *Euglossa melanotricha* (n = 30 families) monitored in the present study, including the reproductive status of the females, their body size (intertegular distance) and ovarian activation (length of the basal oocytes).

| **Nest/Family** |  | **Agressive acts** |  | **Oophagy** |  | **Reproductive status** |  | **Intertegular Distance (mm)** |  | **Basal oocytes (mm)** | | |
| --- | --- | --- | --- | --- | --- | --- | --- | --- | --- | --- | --- | --- |
|  |  |  |  |  |  |  |  |  |  | **First** | **Second** | **Total** |
| N1F1 |  | 59 |  | 10 |  | Dominant |  | 3.57 |  | 2.26 | 1.01 | 3.27 |
|  |  | 0 |  | 0 |  | Subordinate |  | 3.49 |  | 2.23 | 1.03 | 3.26 |
| N1F2 |  | 74 |  | 5 |  | Dominant |  | 3.54 |  | 2.24 | 1.03 | 3.27 |
|  |  | 0 |  | 0 |  | Subordinate |  | 3.62 |  | 2.23 | 0.99 | 3.22 |
| N2F1 |  | 68 |  | 8 |  | Dominant |  | 3.21 |  | 2.21 | 0.89 | 3.10 |
|  |  | 0 |  | 0 |  | Subordinate |  | 3.33 |  | 2.25 | 0.91 | 3.16 |
| N2F2 |  | 49 |  | 7 |  | Dominant |  | 3.27 |  | 2.25 | 0.87 | 3.12 |
|  |  | 0 |  | 0 |  | Subordinate |  | 3.44 |  | 2.24 | 0.91 | 3.15 |
| N2F3 |  | 75 |  | 6 |  | Dominant |  | 3.28 |  | 1.81 | 0.85 | 2.66 |
|  |  | 0 |  | 0 |  | Subordinate |  | 3.18 |  | 1.79 | 0.81 | 2.60 |
| N3F1 |  | 59 |  | 6 |  | Dominant |  | 3.59 |  | 2.27 | 1.05 | 3.32 |
|  |  | 0 |  | 0 |  | Subordinate |  | 3.33 |  | 2.25 | 0.98 | 3.23 |
| N4F1 |  | 42 |  | 7 |  | Dominant |  | 3.42 |  | 2.24 | 0.95 | 3.19 |
|  |  | 0 |  | 0 |  | Subordinate |  | 3.36 |  | 2.21 | 0.89 | 3.10 |
| N5F1 |  | 74 |  | 5 |  | Dominant |  | 3.45 |  | 2.26 | 0.94 | 3.2 |
|  |  | 0 |  | 0 |  | Subordinate |  | 3.39 |  | 2.23 | 0.91 | 3.14 |
| N6F1 |  | 36 |  | 5 |  | Dominant |  | 3.51 |  | 2.27 | 0.99 | 3.26 |
|  |  | 0 |  | 0 |  | Subordinate |  | 3.47 |  | 2.24 | 0.97 | 3.21 |
| N6F2 |  | 77 |  | 6 |  | Dominant |  | 3.32 |  | 2.27 | 0.95 | 3.22 |
|  |  | 0 |  | 0 |  | Subordinate |  | 3.17 |  | 2.25 | 0.88 | 3.13 |
| N6F3 |  | 44 |  | 5 |  | Dominant |  | 4.11 |  | 2.51 | 1.11 | 3.62 |
|  |  | 0 |  | 0 |  | Subordinate |  | 3.71 |  | 2.41 | 1.05 | 3.46 |
| N6F4 |  | 44 |  | 5 |  | Dominant |  | 3.76 |  | 2.53 | 1.01 | 3.54 |
|  |  | 0 |  | 0 |  | Subordinate |  | 3.67 |  | 2.44 | 1.03 | 3.47 |
| N7F1 |  | 55 |  | 5 |  | Dominant |  | 3.89 |  | 2.53 | 1.09 | 3.62 |
|  |  | 0 |  | 0 |  | Subordinate |  | 3.44 |  | 2.37 | 0.99 | 3.36 |
| N7F2 |  | 26 |  | 9 |  | Dominant |  | 3.61 |  | 2.45 | 1.05 | 3.50 |
|  |  | 0 |  | 0 |  | Subordinate |  | 3.59 |  | 2.36 | 0.98 | 3.34 |
| N7F3 |  | 30 |  | 6 |  | Dominant |  | 3.61 |  | 2.44 | 1.03 | 3.47 |
|  |  | 0 |  | 0 |  | Subordinate |  | 3.57 |  | 2.35 | 1.01 | 3.36 |
| N8F1 |  | 71 |  | 5 |  | Dominant |  | 3.21 |  | 2.11 | 0.89 | 3.00 |
|  |  | 0 |  | 0 |  | Subordinate |  | 3.16 |  | 1.89 | 0.91 | 2.80 |
| N8F2 |  | 81 |  | 6 |  | Dominant |  | 3.56 |  | 2.33 | 1.03 | 3.36 |
|  |  | 0 |  | 0 |  | Subordinate |  | 3.61 |  | 2.36 | 1.06 | 3.42 |
| N9F1 |  | 71 |  | 4 |  | Dominant |  | 4.12 |  | 2.55 | 1.13 | 3.68 |
|  |  | 0 |  | 0 |  | Subordinate |  | 3.89 |  | 2.49 | 1.08 | 3.57 |
| N9F2 |  | 45 |  | 5 |  | Dominant |  | 4.14* |  | 2.59 | 1.15 | 3.74 |
|  |  | 0 |  | 0 |  | Subordinate |  | 3.21 |  | 2.13 | 1.01 | 3.14 |
| N10F1 |  | 29 |  | 4 |  | Dominant |  | 4.03* |  | 2.68 | 1.15 | 3.83 |
|  |  | 0 |  | 0 |  | Subordinate |  | 3.57 |  | 2.47 | 1.04 | 3.51 |
| N10F2 |  | 35 |  | 5 |  | Dominant |  | 4.16* |  | 2.63 | 1.13 | 3.76 |
|  |  | 0 |  | 0 |  | Subordinate |  | 3.45 |  | 2.22 | 0.99 | 3.21 |
| N10F3 |  | 24 |  | 4 |  | Dominant |  | 4.07* |  | 2.65 | 1.11 | 3.76 |
|  |  | 0 |  | 0 |  | Subordinate |  | 3.19 |  | 1.94 | 0.97 | 2.91 |
| N10F4 |  | 34 |  | 5 |  | Dominant |  | 4.15* |  | 2.64 | 1.12 | 3.76 |
|  |  | 0 |  | 0 |  | Subordinate |  | 3.23 |  | 1.97 | 0.89 | 2.86 |
| N11F1 |  | 33 |  | 5 |  | Dominant |  | 3.29 |  | 1.89 | 0.91 | 2.80 |
|  |  | 0 |  | 0 |  | Subordinate |  | 3.22 |  | 1.86 | 0.87 | 2.73 |
| N11F3 |  | 35 |  | 4 |  | Dominant |  | 3.33 |  | 2.27 | 0.96 | 3.23 |
|  |  | 0 |  | 0 |  | Subordinate |  | 3.44 |  | 2.29 | 0.98 | 3.27 |
| N12F2 |  | 35 |  | 4 |  | Dominant |  | 3.63 |  | 2.41 | 1.05 | 3.46 |
|  |  | 0 |  | 0 |  | Subordinate |  | 3.61 |  | 2.43 | 1.01 | 3.44 |
| N12F3 |  | 27 |  | 5 |  | Dominant |  | 3.56 |  | 2.46 | 1.03 | 3.49 |
|  |  | 0 |  | 0 |  | Subordinate |  | 3.67 |  | 2.48 | 1.05 | 3.53 |
| N13F2 |  | 33 |  | 4 |  | Dominant |  | 3.54 |  | 2.45 | 1.02 | 3.47 |
|  |  | 0 |  | 0 |  | Subordinate |  | 3.65 |  | 2.41 | 1.04 | 3.45 |
| N14F2 |  | 27 |  | 6 |  | Dominant |  | 3.63 |  | 2.44 | 1.06 | 3.50 |
|  |  | 0 |  | 0 |  | Subordinate |  | 3.64 |  | 2.37 | 1.03 | 3.40 |
| N14F3 |  | 32 |  | 5 |  | Dominant |  | 3.67 |  | 2.43 | 1.06 | 3.49 |
|  |  | 0 |  | 0 |  | Subordinate |  | 3.69 |  | 2.38 | 1.06 | 3.44 |

*Invaders that replaced the dominant resident

**Table 6** Number of individuals analysed in the families of reused nests of *Euglossa melanotricha*, including the newly emerged adults, immature brood and adult females.

| Nest/family |  | Individuals | | | | | | |
| --- | --- | --- | --- | --- | --- | --- | --- | --- |
|  |  | Newly emerged and immature brood | | |  | Adult females | | Total |
|  |  | Males | | Females |  |  |  |  |
| N1F1 |  | 5 |  | 7 |  | 2 |  | 14 |
| N1F2 |  | 4 |  | 3 |  | 2 |  | 9 |
| N2F1 |  | 4 |  | 5 |  | 2 |  | 11 |
| N2F2 |  | 4 |  | 4 |  | 2 |  | 10 |
| N2F3 |  | 4 |  | 3 |  | 2 |  | 9 |
| N3F1 |  | 3 |  | 4 |  | 2 |  | 9 |
| N4F1 |  | 4 |  | 5 |  | 2 |  | 11 |
| N5F1 |  | 5 |  | 3 |  | 2 |  | 10 |
| N6F1 |  | 3 |  | 2 |  | 2 |  | 7 |
| N6F2 |  | 4 |  | 4 |  | 2 |  | 10 |
| N6F3 |  | 3 |  | 3 |  | 2 |  | 8 |
| N6F4 |  | 5 |  | 6 |  | 2 |  | 13 |
| N7F1 |  | 4 |  | 3 |  | 2 |  | 9 |
| N7F2 |  | 3 |  | 4 |  | 2 |  | 9 |
| N7F3 |  | 5 |  | 7 |  | 2 |  | 14 |
| N8F1 |  | 3 |  | 5 |  | 2 |  | 10 |
| N8F2 |  | 3 |  | 4 |  | 2 |  | 9 |
| N9F1 |  | 3 |  | 5 |  | 2 |  | 10 |
| N9F2 |  | 4 |  | 4 |  | 2 |  | 10 |
| N10F1 |  | 5 |  | 8 |  | 2 |  | 15 |
| N10F2 |  | 4 |  | 8 |  | 2 |  | 14 |
| N10F3 |  | 6 |  | 6 |  | 2 |  | 14 |
| N10F4 |  | 4 |  | 5 |  | 2 |  | 11 |
| N11F2 |  | 4 |  | 4 |  | 2 |  | 10 |
| N11F3 |  | 5 |  | 4 |  | 2 |  | 11 |
| N12F2 |  | 3 |  | 4 |  | 2 |  | 9 |
| N12F3 |  | 4 |  | 4 |  | 2 |  | 10 |
| N13F2 |  | 4 |  | 5 |  | 2 |  | 11 |
| N14F2 |  | 3 |  | 4 |  | 2 |  | 9 |
| N14F3 |  | 4 |  | 6 |  | 2 |  | 12 |
| Total |  | 159 |  | 194 |  | 72 |  | 425 |

**Table 7** Microsatellites, primer sequences, hybridization temperatures (HT), and their respective references. F: forward; R: reverse.

| Microsatellites |  | Primer sequence (5' → 3') |  | TH (°C) |  | Reference |
| --- | --- | --- | --- | --- | --- | --- |
| Egc 17 |  | F: AGAGGAGGGTCCAGGAAAGA |  | 54 |  | Souza et al 2007 |
|  |  | R: ACCAGGCGTTCTCATCAAGT |  |  |  |  |
|  |  |  |  |  |  |  |
| Egc 18 |  | F: TGATACAGGTCGGCGTAAAA |  | 58 |  | Souza et al 2007 |
|  |  | R: GGTAACTCCGTCGCGAACTA |  |  |  |  |
|  |  |  |  |  |  |  |
| Egc 24 |  | F: AGGAGAACCGAACAGCGATA |  | 56 |  | Souza et al 2007 |
|  |  | R: CCGAGCTTTTTCTTCCCTCT |  |  |  |  |
|  |  |  |  |  |  |  |
| Egc 26 |  | F: GCCGAACAAACATCTCGTCT |  | 56 |  | Souza et al 2007 |
|  |  | R: CGGAACTTTGATATCGTCGAG |  |  |  |  |
|  |  |  |  |  |  |  |
| Egc 35 |  | F: GATCACGAAACGGTGTAAAGTC | | 54 |  | Souza et al 2007 |
|  |  | R: CTTTCTTCGGATTCGGACTG |  |  |  |  |
|  |  |  |  |  |  |  |
| Egc 37 |  | F: ATCCTCCTCTTCGTGGTCCT |  | 56 |  | Souza et al 2007 |
|  |  | R: GGCAAAACTTCCGCTTGATA |  |  |  |  |
|  |  |  |  |  |  |  |
| Egc 51 |  | F: GGAATGGGATTCATTCAGCA |  | 54 |  | Souza et al 2007 |
|  |  | R: CGAAAGGTCGACAAACCAAT |  |  |  |  |
|  |  |  |  |  |  |  |
| Ann03 |  | F: GATCAGGAATCCCCAAAACG |  | 60 |  | Paxton et al 2009 |
|  |  | R: CATAATCGTGCAACGTTCCTT |  |  |  |  |
|  |  |  |  |  |  |  |
| Ann04 |  | F: CGCGCCCATTCTATTTACAG |  | 54 |  | Paxton et al 2009 |
|  |  | R: GCGCTATCTCCGAATCATTG |  |  |  |  |
|  |  |  |  |  |  |  |
| Ann24 |  | F: GTCGGACATTAGGGTGATGG |  | 56 |  | Paxton et al 2009 |
|  |  | R: TCCGTTTGAATCTGTGGTCTC |  |  |  |  |
|  |  |  |  |  |  |  |
| Ann37 |  | F: CGTTCGAAGGAGAGAACGAC  R: CGGGATACAGGAAAGGAGAG |  | 56 |  | Paxton et al 2009 |

**Video legends**

**Supplementary video 1. Dominant attacking a subordinate female**

**Supplementary video 2. Dominant removing and eating a subordinate’s laid egg**
